# Exploring the Causal Relationship Between Inflammatory Cytokines and Joint Pain: A Mendelian Randomization Study

**DOI:** 10.1155/prm/1958574

**Published:** 2026-02-23

**Authors:** Zehui Yan, Yujia Xi, Changjiang Mu, Jingkai Di, Zijian Guo, Shuai Chen, Chuan Xiang

**Affiliations:** ^1^ Department of Orthopedic, Second Hospital of Shanxi Medical University, Taiyuan, China, sxmu.edu.cn; ^2^ Department of Urology, The Second Hospital of Shanxi Medical University, Taiyuan, China, sxmu.edu.cn

**Keywords:** inflammatory cytokines, joint pain, Mendelian randomization, single nucleotide polymorphisms

## Abstract

**Background:**

Joint pain is a major cause of chronic disability worldwide, with complex mechanisms and limited treatment options. Inflammatory cytokines have been implicated in the pathogenesis of joint pain; however, their causal roles remain unclear. This study employed Mendelian randomization (MR) to explore the causal relationships between 41 inflammatory cytokines and the risk of joint pain.

**Methods:**

We integrated genome‐wide association study (GWAS) summary statistics from European‐ancestry populations, including data on joint pain (1451 cases and 461,559 controls) and inflammatory cytokine levels (8293 Finnish participants). Genetic variants meeting instrumental variable assumptions (*p* < 1 × 10^−5^) were selected. Causal estimates were derived using inverse‐variance weighted (IVW), weighted median, and MR‐Egger regression. Sensitivity analyses incorporated Cochran’s Q test, MR‐Egger intercept analysis, and leave‐one‐out validation to evaluate heterogeneity and potential pleiotropy.

**Results:**

Cutaneous T‐cell attracting chemokine (CTACK)/CCL27 (OR: 0.998 and 95% CI: 0.996–0.999) and interleukin‐2 receptor *α* subunit (IL‐2RA) (OR: 0.997 and 95% CI: 0.995–0.999) were inversely associated with the risk of joint pain, while interleukin‐18 (IL‐18) (OR: 1.0007 and 95% CI: 1.0001–1.0012) demonstrated a positive causal relationship. Sensitivity analyses supported the robustness of the main findings (Cochran’s Q *p* = 0.413), though evidence of horizontal pleiotropy was detected (MR‐Egger intercept *p* < 0.05).

**Conclusion:**

This study identifies IL‐18, CTACK, and IL‐2RA as potential causal mediators of joint pain, providing genetic evidence that informs future precision approaches to analgesia. Future research should validate these findings across diverse populations and elucidate the molecular mechanisms underlying them to advance targeted therapies.

## 1. Introduction

Joint pain, characterized by discomfort, swelling, redness, warmth, stiffness, and limited range of motion in one or more joints, represents a major cause of chronic disability worldwide [1]. Osteoarthritis (OA), the most prevalent skeletal disorder, is a leading contributor to persistent pain and reduced quality of life [2]. While the etiology of pain varies across individuals encompassing trauma, infection, inflammation, autoimmune diseases, and cancer, the underlying mechanisms remain poorly understood, complicating therapeutic strategies [3–5]. Notably, a subset of patients exhibits refractory pain or episodic exacerbations despite existing treatments [6–9], underscoring the need for multitarget interventions. Emerging evidence implicates inflammatory cytokines as key mediators linking joint pathology to pain perception. Proinflammatory factors such as interleukin‐6 (IL‐6), IL‐1β, and tumor necrosis factor‐alpha (TNF‐α) are elevated in OA and may activate nociceptors through direct or indirect pathways [10]. Moreover, cytokines such as C‐reactive protein (CRP) correlate with systemic inflammation and pain severity in OA [11]. Additionally, several other inflammatory factors have been reported to be associated with pain‐related disorders in previous studies. However, most research has focused on disease‐specific associations (e.g., knee OA), leaving the direct causal role of cytokines in joint pain unresolved. Although various theories exist to explain the development of joint pain, further rigorous studies are required to establish a causal relationship between inflammatory factors and joint pain.

Mendelian randomization (MR) is a genetic epidemiological approach that utilizes single‐nucleotide polymorphisms (SNPs) as instrumental variables (IVs) to infer causal relationships between exposures, such as cytokines, and outcomes, including joint pain [12]. This method effectively reduces confounding biases commonly present in observational studies [13–15]. In this study, we applied MR to evaluate the potential causality between 41 inflammatory biomarkers and joint pain using summary statistics from genome‐wide association studies (GWASs).

## 2. Materials and Methods

This MR analysis utilized published GWAS summary statistics. Ethical approval for the original GWASs was obtained from the respective institutional review boards, with informed written consent provided by all participants. No additional ethical approval or participant consent was required for the current analysis. The study was conducted and reported in accordance with the STROBE‐MR guidelines, and a completed checklist is provided as supporting information (Supporting Table 1) [13].

### 2.1. Study Design

A two‐sample MR analysis was performed using publicly available GWAS summary statistics. Genetic instruments for the inflammatory cytokines were selected in accordance with the following three core assumptions of MR: The genetic variants are robustly associated with the exposure (inflammatory cytokines). The genetic variants are not associated with any known or potential confounders. The genetic variants influence the outcome (joint pain) exclusively through the exposure, with no direct or alternative pathways.

### 2.2. Selection of IVs

To identify eligible SNPs as IVs for investigating the relationship between inflammatory cytokines and joint pain, the following quality control steps were applied. First, since applying a strict genome‐wide significance threshold (*p* < 5 × 10^−8^) yielded an insufficient number of SNPs for some cytokines, a more lenient threshold (*p* < 1 × 10^−5^) was adopted to ensure adequate instrument strength. The strength of each included SNP was evaluated by calculating the proportion of variance explained (*R* ^2^) and the *F*‐statistic to avoid weak instrument bias. All selected SNPs exhibited an *F*‐statistic > 10, confirming their suitability as strong instruments.

Potential pleiotropic effects were assessed by querying all candidate SNPs in the PhenoScanner database to identify associations with known confounders of joint pain. SNPs showing significant associations with such confounders were excluded. The final set of instruments demonstrated no substantial evidence of association with major potential confounding factors.

To validate the direction of causality between inflammatory cytokines (exposure) and joint pain (outcome), Steiger directionality testing was performed. Linkage disequilibrium (LD) bias was minimized by clustering SNPs within a 10,000 kb window at an *r* ^2^ threshold < 0.001, and palindromic SNPs were removed. To detect and adjust for horizontal pleiotropy, the MR‐Egger intercept test and the MR‐PRESSO global test were applied for outlier identification and correction [16, 17].

### 2.3. Data Source

The present two‐sample MR analysis utilized publicly available GWAS summary statistics from two independent sources.

Genetic instruments for exposure to inflammatory cytokines were derived from the work of Ahola‐Olli et al., which identified genetic variants associated with circulating levels of 41 cytokines and growth factors. Cytokine concentrations were quantified in plasma or serum samples from 8293 Finnish individuals using a multiplex immunoassay platform. For GWAS, raw cytokine levels were adjusted for age and sex, followed by inverse rank normalization to satisfy model assumptions.

Outcome data on joint pain were obtained from a meta‐analysis of European‐ancestry participants, in which the phenotype was defined based on self‐reported questionnaire responses and/or relevant ICD codes from national health registries. The dataset included 1451 cases and 461,559 controls [18].

### 2.4. Statistical Analysis

Four basic methods were used to explore the causal relationship between different inflammatory factors and joint pain, including the inverse‐variance weighted (IVW) [19], weighted median number (WM) [20], MR‐Egger [19], and the Wald ratio [21]. The Wald ratio is specifically applicable to analyze a single SNP. In the absence of pleiotropy, IVW estimates are considered the primary estimate because IVW can provide a relatively stable and accurate causal assessment by using the meta‐analysis combined with the Wald estimate of each IV [22]. In the absence of horizontal pleiotropy but with potential heterogeneity, the weighted median method was employed to provide robust causal estimates. When multiple IVs were available, results from the MR‐Egger method were prioritized to account for potential pleiotropic effects. Appropriate MR methods were selected based on the number of valid instruments, and sensitivity analyses were conducted for each exposure‐outcome pair [19].

To assess the robustness of the findings, we performed several sensitivity analyses, including the MR‐Egger intercept test, Cochran’s Q test, funnel plots, and leave‐one‐out analysis. Horizontal pleiotropy was evaluated using the intercept from MR‐Egger regression, with a *p* value < 0.05 indicating its presence. Heterogeneity among IVs was examined using Cochran’s Q test (*p* < 0.05 considered significant). Leave‐one‐out analysis was conducted by iteratively removing each SNP associated with the exposure and repeating the IVW analysis to evaluate the influence of individual variants on the overall results.

Given the multiple testing burden inherent in evaluating 41 cytokines, we applied stringent statistical corrections to the primary associations. A Bonferroni‐corrected threshold (*p* < 0.0012, i.e., 0.05/41) was used to adjust for family‐wise error rate, and the Benjamini–Hochberg procedure was additionally employed to control the false discovery rate (FDR).

All statistical analyses were performed using *R* software (Version 4.3.0). MR analyses were conducted primarily with the TwoSampleMR package (Version 0.5.6). IVs were selected and validated using the package’s integrated functions, with potential pleiotropic associations assessed via harmonization with the PhenoScanner database.

## 3. Results

A total of 41 inflammatory cytokines were included in the present study based on their genetically predicted association with joint pain. The number of SNPs serving as IVs for each cytokine ranged from one to seven.

Of the 41 systemic inflammatory regulators examined, only three exhibited genetic variants reaching genome‐wide significance (*p* < 5 × 10^−8^). For the remaining cytokines, a more lenient threshold (*p* < 5 × 10^−6^) was applied to ensure an adequate number of SNPs for subsequent MR analysis.

**FIGURE 1:**
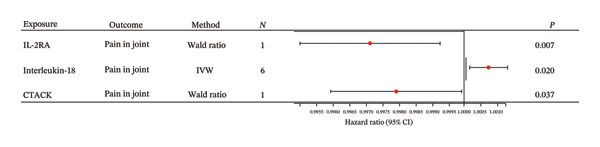
Forest plot of MR estimates for 41 cytokines.

The main findings from the primary MR analysis are summarized as follows. Using the Wald ratio method, genetically predicted levels of cutaneous T‐cell attracting chemokine (CTACK) were inversely associated with the risk of joint pain (OR: 0.998; 95% CI: 0.996–0.999; *p* = 0.037). Similarly, higher levels of interleukin‐2 receptor *α* subunit (IL‐2RA) showed a protective effect (OR: 0.997 and 95% CI: 0.995–0.999; *p* = 0.001) (Figure 1).

After applying strict Bonferroni correction (*p* < 0.0012), only the inverse association between IL‐2RA and joint pain remained statistically significant. Using the less conservative FDR correction (Benjamini–Hochberg method), the association for IL‐2RA remained significant (FDR‐adjusted *p* < 0.05), whereas the association for interleukin‐18 (IL‐18) was attenuated and did not reach the formal significance threshold. The association for CTACK did not survive correction under either method.

**FIGURE 2:**
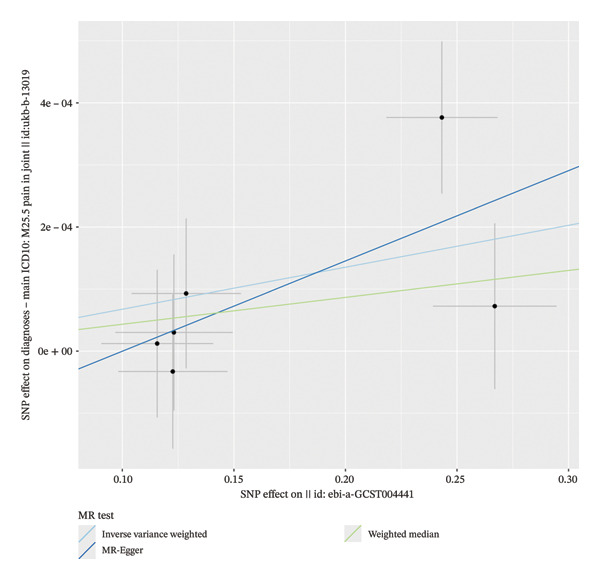
Scatter plots of two‐sample MR analysis of IL‐18 and pain in joint.

**FIGURE 3:**
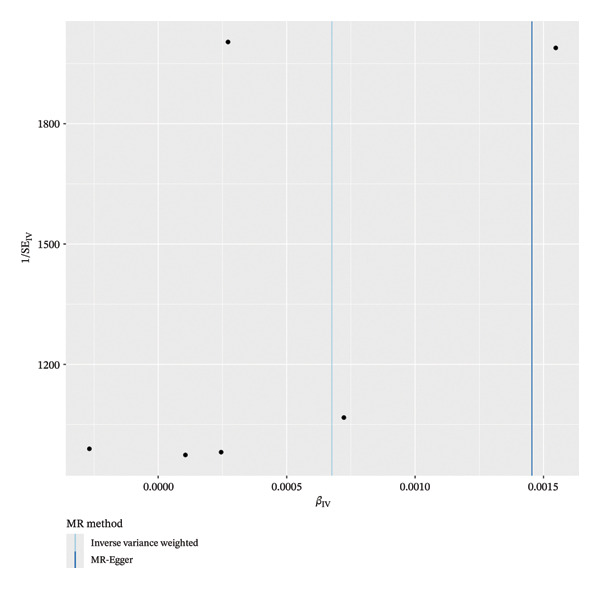
Funnel plots were used to visualize IVs’ sensitivity.

**FIGURE 4:**
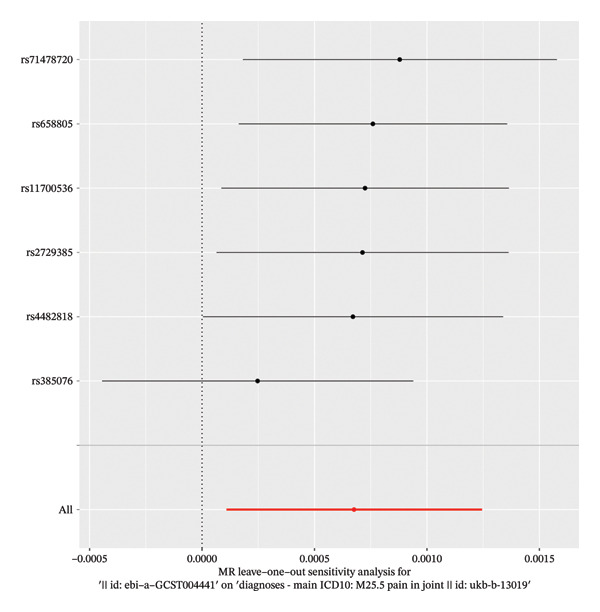
Results of the leave‐one‐out in MR analysis were used to determine the stability of the results.

Cochran’s Q test indicated no significant heterogeneity among the IVs for the analyzed cytokines (*p* = 0.413), suggesting that the observed variation in causal estimates was consistent with chance. The MR‐Egger intercept test indicated the presence of potential horizontal pleiotropy across the full set of 41 cytokines (intercept *p* < 0.05) (Figure 2). However, when examining the three significant exposures individually—CTACK, IL‐2RA, and IL‐18—the funnel plots showed symmetry (Figure 3). Leave‐one‐out sensitivity analysis confirmed that the causal estimate for IL‐18 was not driven by any SNP (Figure 4). Together, these findings suggest that while pleiotropy may be a consideration in the broader screening context, its influence on the three primary causal associations appears limited. The robustness of the IL‐18 result is further supported by the leave‐one‐out analysis, which showed no disproportionate effect of any individual SNP on the overall causal estimate (Figure 4).

## 4. Discussion

In this two‐sample MR analysis, we examined the potential causal effects of 41 inflammatory biomarkers—including growth factors, ILs, and chemokines—on joint pain. Our results indicate that CTACK, IL‐2RA, and IL‐18 may act as upstream regulators in the development of joint pain, suggesting these biomarkers could play active roles in its pathogenesis.

To date, numerous studies have explored associations between various inflammation‐related cytokines and joint pain, often focusing on their utility as diagnostic markers. Previous research has proposed that elevated levels of certain cytokines may contribute to the mechanisms underlying chronic arthralgia. Moreover, the persistent elevation of specific cytokines in chronic joint pain may reflect a compensatory anti‐inflammatory response aimed at modulating inflammatory activity [23].

CTACK is a skin‐specific chemokine expressed by keratinocytes that mediates the migration of lymphocytes to the skin by binding to CCR10 [24]. It has been intensively studied in skin diseases, including atopic dermatitis (AD) and psoriasis [25, 26]. In addition to skin diseases, CTACK plays a role in angiogenesis within tumors, proliferation and dissemination of tumor cells, and cooperates with VEGF to promote lymphangiogenesis [27, 28]. Furthermore, observational studies have found elevated CTACK levels in patients with idiopathic pulmonary fibrosis and suggested it as a predictor of disease outcome [29]. Research on CTACK in the context of autoimmune diseases remains limited. Notably, an in vitro study demonstrated that CTACK/CCL27 significantly inhibited the migration of human subchondral mesenchymal progenitor cells toward microfracture defect sites in synovial fluid, suggesting a mechanism that could potentially compromise cartilage repair and adversely affect the prognosis of patients with rheumatoid arthritis [30]. Elevated concentrations of CTACK/CCL27 have been documented in patients with systemic sclerosis, neuromyelitis optic, and idiopathic inflammatory myopathy [31–33]. However, to our knowledge, no prior studies have reported a direct association between CTACK/CCL27 levels and joint pain. Although CTACK has been extensively studied as a skin‐specific chemokine, its direct link to joint pain is, to our knowledge, first proposed here at the genetic level. CTACK is known to guide immunosuppressive T cells to inflammatory sites by binding to its cognate receptor CCR10. We speculate that CTACK might indirectly alleviate pain by recruiting regulatory T cells (Tregs) to suppress excessive local inflammation within the joints [34]. Additionally, studies indicate that CCR10 is expressed on certain sensory neurons, suggesting that CTACK could also directly modulate neuronal excitability through this receptor, thereby producing analgesic effects akin to those of some other chemokines. Future research should prioritize detecting upregulation of the CTACK–CCR10 axis in the synovial tissue of patients with joint pain and employing animal models to validate its role in pain regulation.

ILs are a class of cytokines produced by and acting on a variety of cells [35]. Currently, approximately 40 distinct ILs have been identified in humans. Interleukin‐2 (IL‐2) plays a crucial role in the immune system, particularly in regulating T‐cell proliferation and activation [36]. Its soluble receptor, IL‐2RA, modulates this pathway by competitively binding IL‐2, thereby inhibiting excessive T‐cell activation and helping to maintain immune homeostasis and suppress autoimmune responses [36]. However, IL‐2RA inhibits the activation and proliferation of T cells by blocking the IL‐2 signaling pathway, thus maintaining the immune homeostasis and suppressing the autoimmune response [37], Due to its central role in immune regulation, IL‐2RA has emerged as a therapeutic target in autoimmune diseases and organ transplantation [38]. Prior studies suggest that IL‐2RA may serve as a potential biomarker for arthritis development in patients with seropositive arthralgia [39] and could be linked to pain perception in rheumatoid arthritis [40]. Nevertheless, the specific role of IL‐2RA in joint pain remains incompletely understood, warranting further investigation.

IL‐18, another member of the interleukin family, is a potent proinflammatory cytokine involved in host defense against infections and in the regulation of both innate and adaptive immune responses [41].

IL‐18 plays a significant role in both innate and adaptive immunity, implicating it in the pathogenesis of various inflammatory and autoimmune diseases [42]. Evidence suggests that IL‐18 levels are associated with osteoporosis and may contribute to rheumatoid arthritis, conditions frequently accompanied by joint pain. As early as 2008, studies in mice demonstrated that inhibiting the IL‐18 signaling pathway with anti‐IL‐18 antibodies could attenuate nerve injury‐induced tactile allodynia, positioning IL‐18 as an important pronociceptive factor based on immunohistological findings [43]. Joint pain predominantly affects the elderly, most commonly due to OA [10]. Persistent nociceptive input from an OA joint is believed to sensitize peripheral and central nervous pathways, thereby amplifying pain perception [44]. Inflammatory cytokines, including IL‐18, are thought to exacerbate this process. Some studies indicate that IL‐18 can aggravate bone and joint pain through pyroptosis‐related pathways [45]. For instance, caspase‐1 activation via the NLRP3 inflammasome in macrophages promotes the release of proinflammatory cytokines such as IL‐1β and IL‐18 and induces pyroptosis, which may enhance nociceptive signaling in joint tissues—particularly in synovium—a phenomenon often described as “hyperalgesia” [46]. Furthermore, IL‐18 stimulation has been shown to induce autophagy defects through the PI3K/Akt/mTOR pathway, leading to degradation of chondrocyte‐specific genes and potentially contributing to pain [47]. The resulting cartilage and joint tissue destruction may facilitate excessive sensory and sympathetic innervation, further amplifying pain sensation. Supporting this mechanistic view, blockade of IL‐18 signaling has been reported to reduce neuropathic pain and enhance the efficacy of opioid analgesics such as morphine and buprenorphine [48], which aligns indirectly with our genetic findings.

However, the evidence remains somewhat contradictory. One study reported that significantly elevated levels of IL‐18 mRNA and protein were not detected in patients with osteoarthritic pain but were observed in the synovial tissue of age‐matched patients with rheumatoid arthritis [49].

These findings collectively suggest that IL‐18 levels may serve as a potential contributor to bone and joint pain. Serum IL‐18 has been proposed as a promising diagnostic biomarker for adult‐onset Still’s disease in patients presenting with joint pain, with measurable levels observed during both acute and recovery phases [50]. To date, this represents one of the limited clinical studies directly linking IL‐18 to joint pain. Further investigation is needed to clarify the prognostic and diagnostic utility of IL‐18, as well as its mechanistic role in joint pain, building upon existing evidence and the genetic insights provided by the present MR study.

Therapeutic strategies targeting IL‐18 have already entered clinical development, offering a viable pathway for translating our genetic findings into potential patient benefit. For instance, recombinant human IL‐18 binding protein (tadekinig alfa) and neutralizing anti‐IL‐18 antibodies have been evaluated in clinical trials for autoimmune diseases and certain cancers. Our study provides a genetic rationale for considering the inclusion of patients with chronic joint pain—particularly those with elevated IL‐18 biomarkers—in such therapeutic trials. We recommend that future clinical research prioritize investigating the efficacy and safety of IL‐18 inhibitors in individuals with refractory joint pain and evidence of IL‐18 pathway activation. This approach represents a pivotal step toward achieving precision analgesic therapy for joint pain.

These findings carry several important clinical implications. First, they highlight novel therapeutic targets for the more effective management of joint pain. Current first‐line analgesics such as nonsteroidal anti‐inflammatory drugs (NSAIDs) often lack specificity and demonstrate limited efficacy in a substantial proportion of patients [51]. The inflammatory mediators identified here could be selectively modulated, offering the potential to ameliorate immune‐mediated pathology without inducing broad immunosuppression.

To our knowledge, this is the first MR study to systematically assess the causal relationship between joint pain and 41 inflammatory cytokines. Nevertheless, several limitations should be considered. First, MR analyses are subject to inherent assumptions, and residual confounding or pleiotropy may influence causal estimates. Second, as this study relied on publicly available GWAS summary statistics, detailed demographic and clinical information at the individual level was unavailable, precluding subgroup or stratified analyses. Third, the populations included in the source GWAS were exclusively of European ancestry; therefore, caution is warranted when generalizing these findings to other ethnic groups. Fourth, although multiple comparison corrections were applied, the exploratory screening of 41 cytokines means that the identified associations—particularly for IL‐18 and CTACK—should be interpreted as hypothesis generating. Future studies with predefined hypotheses are required to confirm these links. Finally, while the two‐sample MR design utilized data from independent cohorts and sensitivity analyses supported robustness, potential sample overlap in the underlying populations cannot be entirely excluded, which may lead to a modest overestimation of instrument strength. Further validation in independent datasets and translational studies is needed before these findings can inform clinical diagnosis or therapeutic strategies.

In this two‐sample MR analysis, we assessed the causal roles of 41 circulating biomarkers—including growth factors, interleukins, and chemokines—in joint pain. Our results suggest that CTACK, IL‐2RA, and IL‐18 may act as upstream regulators in the development of joint pain. While the associations of CTACK and IL‐2RA with joint pain have been relatively understudied, their identification here underscores their potential importance in pain pathogenesis, warranting further investigation. In contrast, IL‐18 appears to hold particular clinical relevance, potentially serving as an upstream diagnostic indicator whose levels—measurable in both acute and recovery phases—could aid in predicting joint pain progression. Collectively, these findings offer novel diagnostic insights and highlight specific inflammatory pathways that may be targeted to develop more effective therapeutic and preventive strategies for joint pain.

## 5. Conclusion

This MR study provides genetic evidence supporting a causal role for CTACK, IL‐2RA, and IL‐18 in the risk of joint pain. Specifically, the association identified for IL‐18 strengthens its rationale as a promising candidate biomarker, warranting further clinical validation. The signals for CTACK and IL‐2RA highlight novel biological pathways that merit deeper mechanistic investigation. Future research should focus on validating these associations in diverse populations and elucidating the underlying molecular mechanisms to inform the development of targeted therapeutic strategies.

## Author Contributions

Zehui Yan and Yujia Xi contributed equally to this work. All authors contributed to the research concept. Zehui Yan and Yujia Xi were responsible for the design the study and writing manuscript. Changjiang Mu, Jingkai Di, Zijian Guo, and Shuai Chen contributed to data acquisition and analysis. Chuan Xiang revised the manuscript.

## Funding

No funding was received for this manuscript.

## Disclosure

All authors read and approved the final manuscript.

## Conflicts of Interest

The authors declare no conflicts of interest.

## Supporting Information

The document is the STROBE‐MR (Mendelian Randomization Research Epidemiological Observational Research Report Specification) checklist. It comprehensively lists the implementation status of the recommended report items in each section of this study, including the title, abstract, introduction, methods, results, and discussion, and marks the corresponding page numbers to ensure that the research complies with internationally recognized academic reporting standards.

## Supporting information


**Supporting Information** Additional supporting information can be found online in the Supporting Information section.

## Data Availability

All the data used in the present study had been publicly available, and the source of the data had been described in the main text and Supporting Information.
